# Microbiome Alterations and Alzheimer’s Disease: Modeling Strategies with Transgenic Mice

**DOI:** 10.3390/biomedicines11071846

**Published:** 2023-06-27

**Authors:** Juan Antonio López-Villodres, Alejandro Escamilla, Silvia Mercado-Sáenz, Carmen Alba-Tercedor, Luis Manuel Rodriguez-Perez, Isabel Arranz-Salas, Raquel Sanchez-Varo, Diego Bermúdez

**Affiliations:** 1 Departamento Fisiologia Humana, Histologia Humana, Anatomia Patologica y Educacion Fisica y Deportiva, Facultad de Medicina, Universidad de Malaga, 29071 Malaga, Spain; jantoniolv@uma.es (J.A.L.-V.); jandromilla@uma.es (A.E.); smercad@uma.es (S.M.-S.); mcalba@uma.es (C.A.-T.); luismrp@uma.es (L.M.R.-P.); iarranz@uma.es (I.A.-S.); dbermudez@uma.es (D.B.); 2 Instituto de Investigacion Biomedica de Malaga-IBIMA-Plataforma Bionand, 29071 Malaga, Spain; 3 Unidad de Anatomia Patologica, Hospital Universitario Virgen de la Victoria, 29010 Malaga, Spain; 4 Centro de Investigacion Biomedica en Red sobre Enfermedades Neurodegenerativas (CIBERNED), 28031 Madrid, Spain

**Keywords:** Alzheimer’s disease, transgenic mouse models, amyloid, tau, microbiota, dysbiosis, metabolic diseases, neuroinflammation, probiotics, fecal microbiota transplantation

## Abstract

In the last decade, the role of the microbiota–gut–brain axis has been gaining momentum in the context of many neurodegenerative and metabolic disorders, including Alzheimer’s disease (AD) and diabetes, respectively. Notably, a balanced gut microbiota contributes to the epithelial intestinal barrier maintenance, modulates the host immune system, and releases neurotransmitters and/or neuroprotective short-chain fatty acids. However, dysbiosis may provoke immune dysregulation, impacting neuroinflammation through peripheral–central immune communication. Moreover, lipopolysaccharide or detrimental microbial end-products can cross the blood–brain barrier and induce or at least potentiate the neuropathological progression of AD. Thus, after repeated failure to find a cure for this dementia, a necessary paradigmatic shift towards considering AD as a systemic disorder has occurred. Here, we present an overview of the use of germ-free and/or transgenic animal models as valid tools to unravel the connection between dysbiosis, metabolic diseases, and AD, and to investigate novel therapeutical targets. Given the high impact of dietary habits, not only on the microbiota but also on other well-established AD risk factors such as diabetes or obesity, consistent changes of lifestyle along with microbiome-based therapies should be considered as complementary approaches.

## 1. Introduction

Alzheimer’s disease (AD) constitutes the most prevalent form of dementia, being considered one of the current global epidemics with more than 55 million people affected worldwide (data from Alzheimer’s Association). Owing to population ageing, together with the lack of effective therapeutic interventions, estimates now suggest that the number of AD patients will reach 150 million by 2050 [1].

AD brains display two main histopathological hallmarks: the buildup of amyloid-beta (Aβ) peptide in the form of extracellular amyloid plaques, and the presence of intraneuronal neurofibrillary tangles (NFTs) formed by hyperphosphorylated tau (phospho-tau). The hippocampus, involved in learning and memory, is among the earliest cerebral areas affected by this disease [2]. To date, the etiology of AD remains unknown. Initially, the amyloid hypothesis was the most accepted theory, stating that Aβ accumulation triggers the sequence of pathological events leading to synaptic loss/dysfunction, neuroinflammation, and neuronal degeneration [3,4]. Nevertheless, many pharmacological approaches based on this amyloid paradigm have systematically failed to meet successful clinical outcomes, highlighting the need for novel theoretical frameworks to develop alternative therapeutical targets [5].

In this sense, compelling evidence points to a multifactorial origin of AD, including aging, sex [6], comorbid conditions (obesity, diabetes, depression, etc.) [7,8], and genetic risk factors (APOE4 allele, genetic polymorphisms, etc.) [9]. In this scenario, the role of microbiota is progressively gaining interest [10,11]. Approximately 100 trillion microorganisms live in the human body, most of them within the gastrointestinal tract (gut microbiota), and those present in the oral cavity (oral microbiota) are also of relevance [12]. When balanced, microbiota constitutes a symbiotic mutualistic relationship with the host, producing beneficial substances such as neurotransmitters [13] or short-chain fatty acids (SCFAs) [14]. On the other hand, dysbiosis or dysbacteriosis may be defined as the imbalance of microbiota, due to either quantitative or qualitative changes in its composition, distribution, or metabolic activities. Dysbiosis and the release of Gram-negative lipopolysaccharide (LPS) and toxic microbial end-products can alter gut permeability, leading to local, systemic, and cerebral inflammation. LPS itself has been detected in AD brains and may contribute to amyloid toxicity [15]. Thus, dysbiosis has been suggested to be linked to the onset and/or progression of psychiatric and neurological diseases such as AD, Parkinson Disease (PD), multiple sclerosis (MS), autism spectrum disorder (ASD), and major depression disorder (MDD), among others [16]. This review focuses on highlighting the value of animal models as tools for improving our current knowledge about the mechanisms involved in connecting dysbiosis with AD neuropathology, and for analyzing potential novel microbiome-based therapeutic strategies.

## 2. Microbiota–Gut–Brain Axis

The term “gut–brain axis” refers to several neuro–immune–endocrine pathways that participate in bidirectional brain–body crosstalk, leading to a more integrative comprehension of human health and diseases. This network includes the signaling mediated by the vagus nerve, the immune system, and the hypothalamus–pituitary–adrenal (HPA) axis [10,17]. More recently, microbiota and their metabolic products were involved in this complex framework. For instance, microbiota produce neuroprotective SCFAs such as butyrate, and are involved in the secretion of neuroactive metabolites, including gamma-aminobutyric acid (GABA), dopamine, serotonin, as well as other tryptophan-derived catabolites [13,18,19,20,21]. Thus, it is reasonable that the former term has finally been extended to “microbiota–gut–brain” (MGB) axis [22,23].

Intestinal microbiota are made not only of bacteria but also a multitude of archaea, viruses, yeasts, unicellular eukaryotes, helminths, and parasites hosted in the gut. In human adults, the bacteria forming microbiota belongs to four main phyla: Firmicutes (51%) and Bacteroidetes (48%), followed by Actinobacteria and Proteobacteria (1%) [24]. The composition of the microbiota may change over the lifespan, influenced by childbirth (natural or cesarean), sex, dietary habits, exercise, chemicals, infections, antibiotics, and many other factors, including metabolic diseases and aging [16,25]. In the last decade, the field of neuroscience has attained a significant increase in the growth rate of knowledge about the microbiome, with a special emphasis on intestinal microbiota, which have been incorporated into the study of a range of neurodegenerative diseases from a holistic paradigm. 

According to the impact of bacterial metabolic end-products and their neuro–immunomodulatory properties on the host, microbiota can be classified into different functional subsets (Table 1):

Immunomodulatory gut microbiota: Several studies have linked gut microbiota to local and systemic regulation of the immune response [26,27,28]. The immunomodulatory microbial subset performs continuous signaling and training of the immune system, enabling the differentiation of commensal from pathogenic microbiota. In general, Gram-positive commensal bacteria play a dominant role in maintaining regulatory T cell (Treg) homeostasis [29], as confirmed by the experiments in which the reconstitution of germ-free (GF) mice with Gram-positive spore-forming microorganisms restored the Treg population [30]. Commensal bacteria such as *Bifidobacterium, Bacteroides fragilis,* and *Clostridium* spp. stimulate antigen presenting cells (APCs) to promote secretion of the anti-inflammatory IL-10 and Treg responses that modulate Natural Killer (NK)-T cells as well as Th1 and Th17 pathways [31]. For instance, butyrate produced by *Clostridium butyricum* mediates this immunomodulatory signaling [32]. On the other hand, alterations in the composition of *Bifidobacterium* and *Lactobacillus* may influence the frequency of mucosal Treg cells, which participate in the development and pathogenesis of different disorders such as the inflammatory bowel disease (IBD) [31] or Graves’ disease [33]. The role of gut microbiota in the efficacy of checkpoint blockade immunotherapy has also been described in cancer research [34].

The immunomodulatory role of *B. fragilis* has been frequently studied [35,36]. For instance, Hidalgo-Cantabrana et al. [37] identified the *B. fragilis*-derived B12 peptide that polarized Th17 and Th22 responses in human peripheral blood mononuclear cells (PBMCs) from healthy controls. *B. fragilis* is also implicated in antiviral responses, together with dendritic cells and interferon-β (IFN-β) [38]. *Akkermansia muciniphila*, a mucus layer degrading bacterium, participates in immunomodulation as well [39]. Specifically, a membrane phospholipid from *A. muciniphila* has been recently reported to mediate immunity homeostasis through a non-canonical TLR1–TLR2 pathway [40]. On the other hand, the increase in *Escherichia/Shigella* ratio leads to peripheric inflammation in many pathological conditions, including AD [41], diabetic nephropathy [42], allergy development [43], or colorectal cancer [44]. 

Not only bacterial agents participate in the host immunomodulation. *Candida albicans* has been shown to interact with mucosal innate immune cells through the pathways associated with Dectin-1 in macrophages [45]. Additionally, *Tritrichomonas musculis*, a commensal protist, elicits inflammasome activation in epithelial cells, leading to the secretion of IL-18 and the downstream activation of the intestinal immune system, including changes in myeloid and innate lymphoid cells. Moreover, protozoan colonization markedly alters epithelial inflammasome signaling and the activation state of the immune system, being a potential source of inter-facility variation [46]. To sum up, several gut microorganisms are involved in immunomodulation, not only in the maintenance of homeostasis but also in the response to several pathogens. The lack of immunomodulatory microbiota under dysbiosis gives rise to chronic intestinal inflammatory processes that may degenerate into increased intestinal permeability (leaky gut syndrome). Given their role in modulating the immune system, this subpopulation of intestinal microbiota should be considered as a therapeutic target in relation to the development of neurodegenerative disorders such as AD. 

Butyrate-producing gut microbiota: Many bacteria can produce SCFAs (acetate, propionate, or butyrate, among others) during the fermentation of dietary fiber [14,47]. These molecules are mainly released to the colon, serving as a substrate for energy metabolism not only to enterocytes but also to other cells, among other functions. In humans, butyrate is produced by Gram-positive Firmicutes, such as *Eubacterium rectale*, *Faecalibacterium prausnitzii*, or *C. butyricum* [32,48]. This SCFA favors gastrointestinal homeostasis by promoting the growth of intestinal epithelial cells and the expression of tight junction proteins, being involved in the maintenance of the intestinal barrier. In addition, butyrate has been positively correlated with endothelial and BBB maintenance [47,49]. Moreover, butyrate stands as a very relevant immunomodulatory, anti-inflammatory, and neuroprotective molecule (for review, see [14,50]). Importantly, SCFAs have been reported to be key molecules in modulating microglia maturation, morphology, and function [51]. Furthermore, the reduction of SCFAs production by microbiota can induce inflammation, leaky gut, and microglial activation in human diseases [52]. For instance, the decrease in the abundance of *E. rectale* is associated with peripheric inflammation in AD patients [41]. Considering all these beneficial properties, SCFAs (and specially butyrate) have been regarded as promising candidate molecules in the context of several neurodegenerative diseases, including AD [14,53]. 

GABA-producing and GABA-consuming intestinal microbiota: GABA is the main inhibitory neurotransmitter of the central nervous system (CNS). GABA is synthesized by glutamate decarboxylase (GAD) from glutamic acid and binds to GABA-A and GABA-B receptors [54]. There are many GABA-producing bacteria in the human gut, including *Bifidobacterium* or *Lactobacillus* genera [55,56]. Intestinal microbiota expressing GAD break down glutamate from food, releasing GABA [56,57] for the reduction of intracellular pH. On the other hand, some bacterial species consume GABA, for example, via the GABA shunt. Therefore, the balance of these microorganisms has an impact on peripheric GABA levels. Then, the penetration of GABA from the periphery into the CNS can be mediated by a specific transporter through the BBB [58]. 

GABA concentrations are altered in dysbiosis, which might partly explain the appearance or progression of diseases such as MDD and AD [59]. In fact, GABAergic dysfunction is associated with cortical network hyperactivity, memory alterations, depression, and AD [60,61]. GABAergic system has been found to be affected in cerebrospinal fluid (CSF) and brain samples from AD patients [54,60,62,63,64] and transgenic models [65,66]. Moreover, depression itself is considered an AD risk factor [8], and MDD patients exhibit gut microbiota alterations such as a reduction in richness and diversity (for review, see [20,67]). In addition, peripheric GABA has been linked to glucose homeostasis and the regulation of insulin sensitivity [68]. Curiously, a very recent work in obese mice has shown that GABA treatment can mediate adipocyte beiging and weight loss and reduce adipose inflammation through microbiota-mediated mechanisms [69]. Thus, the GABA-modulating microbiome seems to interfere with metabolic diseases considered AD risk factors, such as diabetes and obesity. Janik et al. [70] reported that probiotic supplementation with oral *Lactobacillus rhamnosus* (JB-1) to BALB/c healthy mice elicited an increase in GABA cerebral levels detected by magnetic resonance spectroscopy. However, there still are conflicting results about whether or not the rise in peripheric GABA levels impacts cerebral content [13]. Therefore, more studies are needed to unravel the weight of dysbiosis in cerebral GABA dysfunction.

Serotonin metabolism by the intestinal microbiota: The neurotransmitter serotonin (5-hydroxytryptamine, 5-HT) is obtained from the essential amino acid tryptophan. 5-HT functions as a neuroactive molecule either on the enteric nervous system (ENS), controlling intestinal secretion and motility, or regulating emotions and other processes at the level of the CNS. Approximately 90% of human serotonin is produced within the intestine, mainly by enteroendocrine cells modulated via the secretion of SCFAs or secondary bile acids by gut microbiota [71,72]. In fact, GF mice exhibit reduced plasma levels of serotonin [18]. The production of 5-HT by several genera and species, including *Streptococcus* spp., *Escherichia* spp., and *Enterococcus* spp., has also been reported (for review, see [67]). Only the remaining 5–10% of 5-HT is produced within the brain. 

Serotonin regulates mood and cognitive processes; therefore, depressive symptoms, anxiety, and other behavioral alterations can be elicited by serotonergic dysfunction. In fact, this system is affected in AD patients, displaying a reduced content of serotonin in brain and CSF, and a loss of serotonergic receptors in different cerebral regions such as the amygdala or hippocampus. Moreover, the treatment with selective serotonin reuptake inhibitors (SSRI) ameliorates behavioral and cognitive symptoms in AD subjects [73]. Significantly, serotonin conjugated to isoalantolactone inhibited BACE-1, prevented the aggregation of Aβ1-42, and exhibited neuroprotective activity in vitro using SH-SY5Y neuroblastoma cells. Furthermore, this compound improved learning and memory in the 5xFAD mouse model of AD [74]. Given that gut microbiota regulates peripheral serotonin levels, it might be considered as a therapeutic target for serotonergic disturbances in neurodegenerative or neuropsychiatric disorders. 

Microbiota producing other tryptophan-derived metabolites: Several routes compete for tryptophan as the primary substrate in detriment of serotonin production. In the host, the kynurenine (KYN) pathway may take place in the liver or the brain, yielding different metabolites such as kynurenic acid (KYNA) or quinolinic acid (QUIN) [75]. The direct decarboxylation of tryptophan by the intestinal bacteria yields tryptamine [21]. *E. coli* has been reported to express the enzyme aspartate aminotransferase, which converts KYN into KYNA [76]. Such microbial tryptophan-derived metabolites have influence over the intestinal homeostasis, the CNS, and the immune system (for review, see [21,77]). For instance, KYNA exhibits neuroprotective and neuromodulatory properties over the acetylcholine receptor, playing a key role in nociception, neurodegeneration and neuroinflammation. A decrease in KYNA has been detected in AD and PD patients, and intracerebral administration of this metabolite to CFLP mice improved memory [78]. Importantly, some of these molecules from the tryptophan metabolic pathway may function as immunomodulators or Aβ aggregation inhibitors (tryptamine, 5-methoxytryptamine and others). Conversely, the KYN pathway has been associated with intestinal inflammatory processes such as ulcerative colitis [79].

Other downstream metabolites may exert neurotoxic effects, such as QUIN [80]. QUIN is produced from tryptophan by the enzymes tryptophan dioxygenase (TDO) and indole-dioxygenase (IDO) in many tissues in response to immune activation, and it can be produced by microglia as well. IDO enzymatic activity has been associated with increased oxidative stress. Moreover, QUIN functions as an NMDA receptor agonist, with a potent neurotoxic effect that participates in neurodegenerative processes including AD, MS, or PD. In fact, QUIN levels are higher in serum and neurons from post-mortem AD patients than from controls; it has been found within periplaque glial cells and correlates with neuroinflammation (for review, see [75]). Wu et al. [81] reported higher TDO, IDO, and QUIN levels in AD hippocampus compared to healthy controls, as well as a progressive age-associated increase in TDO and QUIN levels in the 3xTg-AD mouse brain. However, authors did not examine a possible link with gut microbiota activity or dysbiosis in this work. Additionally, tryptophan might as well be converted by intestinal bacteria to indole [82] and indole derivatives such as indole-3-acetic acid (IAA) and indole-3-propionic acid (IPA) [21,75,83]. Some indoles display anti-IDO activity and anti-aggregative or anti-inflammatory properties [80]. Thus, gut microbiota related to tryptophan metabolism seems to impact on brain function, contributing to health or disease. Even though there are still conflicting results, dysbacteriosis concerning tryptophan metabolism might participate in the onset or worsening of AD. 

Biogenic amines and other compounds produced by microbiota: Biogenic amines (BAs) are a group of diverse metabolites, including the above-mentioned tryptamine, histamine, tyramine, or cadaverine, that can be found either in food or produced by gut microbiota. Tryptamine can cross the BBB and displays a dose-dependent cytotoxic potential. Moreover, the formation of structures resembling NFTs in SH-SY5Y cells and mouse brains has been observed [84]. Hypertension, a risk factor for cardiovascular diseases and AD, can be produced by tryptamine and other BAs such as tyramine and phenylethylamine [85]. Finally, increased levels of histamine, cadaverine, and other BAs were found by widely targeted metabolomics of post-mortem CSF from AD subjects [86]. Finally, gut-derived trimethylamine N-oxide (TMAO) has been implicated in the development of AD [87,88]. Although the link between TMAO and AD remains unclear, some studies suggest that elevated TMAO in aging might impair cognitive function and aggravate the AD pathology [89]. 

Overall, these data support the contention that an unbalanced microbiome is linked to the onset or progression of many gastrointestinal, metabolic, neuropsychiatric, and neurodegenerative disorders.

**Table 1:** Bacterial classification into functional subsets. Phylum, genera, microbial species, or strains are provided as examples of each subgroup. GABA, Gamma-aminobutyric acid; IAA, indol-3 acetic acid; IPA, indol-3 propionic acid; KYNA, kynurenic acid; QUIN, quinolinic acid; TMA, trimethylamine.

Functional Subset	Microorganism	Phylum	References
Biogenic amine-producing microbiota	*Escherichia coli*	Proteobacteria	[90,91,92]
	*Morganellaceae (Morganella morganii)*	Proteobacteria	
	*Proteus (P. mirabilis, P. vulgaris…)*	Proteobacteria	
	*Pseudomonadaceae*	Proteobacteria	
	*Raoultella*	Proteobacteria	
	*Clostridium perfringens*	Firmicutes	
	*Enterococcus faecalis*	Firmicutes	
	*Lactobacillus vaginalis*	Firmicutes	
	*Staphylococcus*	Firmicutes	
Butyrate-producing microbiota	*Anaerostipes* spp. *(A. butyraticus, A. caccae, A. hadrus…)*	Firmicutes	[48,67,93,94]
	*Butyricicoccus pullicaecorum*	Firmicutes	
	*Clostridium butyricum,* *C. symbiosum*	Firmicutes	
	*Coprococcus comes,* *C. eutactus, C. cactus*	Firmicutes	
	*Eubacterium rectale, E. hallii*	Firmicutes	
	*Faecalibacterium prausnitzii*	Firmicutes	
	*Roseburia faecis, R. hominis, R. intestinalis, R. inulinivorans*	Firmicutes	
GABA-producing microbiota	*Bifidobacterium adolescentis* (DPC6044)	Actinobacteria	[13,55,56,67]
	*Bifidobacterium angulatum* (ATCC27535)	Actinobacteria	
	*Bifidobacterium dentium* (DPC6333)	Actinobacteria	
	*Bifidobacterium infantis* (UCC35624)	Actinobacteria	
	*Lactobacillus brevis* (DPC6108)	Firmicutes	
	*Lactobacillus buchneri* (MS)	Firmicutes	
	*Lactobacillus paracasei* NFRI (7415)	Firmicutes	
	*Lactobacillus plantarum* (ATCC14917)	Firmicutes	
	*Lactobacillus reuteri* (100–23)	Firmicutes	
	*Lactobacillus rhamnosus* (YS9)	Firmicutes	
	*Lactobacillus delbrueckii* subsp. *bulgaricus* (PR1)	Firmicutes	
	*Streptococcus salivarius* subsp. *thermophilus* (Y2)	Firmicutes	
	*Bacteroides* spp.	Bacteroidetes	
	*Parabacteroides* spp.	Bacteroidetes	
	*Alistipes* spp.	Bacteroidetes	
	*Escherichia* spp.	Proteobacteria	
GABA-consuming microbiota	*Klebsiella pneumoniae*	Proteobacteria	[56,95]
	*Pseudomonas genera*	Proteobacteria	
	*Acinetobacter*	Proteobacteria	
	*Mycobacterium*	Actinobacteria	
Immunomodulatory microbiota	*Bifidobacterium* spp. *(B. breve, B. bifidum, B. longum...)*	Actinobacteria	[39,40,96,97]
	*Lactobacillus (L. acidophilus*	Firmicutes	
	*L. gasseri, L. salivarius, L plantarum, L. casei Shirota, L. delbrueckii* subsp. *Bulgaricus, L. helveticus, L. reuteri, L. rhamnosus, L. johnsonii, L. fermentum)*		
	*Bacillus (B. coagulans, B. subtilis)*	Firmicutes	
	*Lactococcus lactis*	Firmicutes	
	*Streptococcus thermophilus*	Firmicutes	
	*Escherichia coli*	Proteobacteria	
	*Akkermansia muciniphila*	Verrucomicrobia	
IAA-producing microbiota	*Bacteroides (B. ovatus, B. fragilis)*	Bacteroidetes	[21,77,98]
	*Parabacteroides distasonis*	Bacteroidetes	
	*Bifidobacterium (B. adolescentes,*	Actinobacteria	
	*B. longum* subsp. *longum,*		
	*B. pseudolongum)*		
	*Clostridium (C. bartlettii,* *C. difficile, C. lituseburense,* *C. paraputrificum,* *C. perfringens, C. putrefaciens, C. saccharolyticum,* *C. sticklandii, C. subterminale*)	Firmicutes	
	*Eubacterium (E. hallii,*	Firmicutes	
	*E. cylindroides)*		
	*Peptostreptococcus asscharolyticus*	Firmicutes	
	*Escherichia coli*	Proteobacteria	
Indole-producing microbiota	*Bacteroides (B. thetaiotaomicron,* *B. ovatus…)*	Bacteroidetes	[21,67,77,82]
	*Clostridium (C. bifermentans,* *C. ghoni, C. limosum,* *C malenomenatum,* *C. lentoputrescens, C. tetani,* *C. tetanomorphum, C. sordellii)*	Firmicutes	
	*Enterococcus faecalis*	Firmicutes	
	*Peptostreptococcus asscharolyticus*	Firmicutes	
	*Fusobacterium nucleatum*	Fusobacteria	
	*Desulfovibrio vulgaris*	Proteobacteria	
	*Escherichia coli*	Proteobacteria	
	*Haemophilus influenza*	Proteobacteria	
IPA-producing microbiota	*Lactobacillus reuteri*	Firmicutes	[21,77,83]
	*Clostridium (C. botulinum,* *C. caloritolerans,* *C. paraputrificum,* *C. sporogenes, C. cadaveris)*	Firmicutes	
	*Peptostreptococcus (P. asscharolyticus, P. sussellii,* *P. anaerobious, P. stomatis)*	Firmicutes	
	*Akkermansia*	Verrucomicrobia	
KYNA-producing microbiota	*Escherichia coli*	Proteobacteria	[67,76,99]
	*Pseudomonas* spp.	Proteobacteria	
	*Lactobacillus* spp.	Firmicutes	
QUIN-producing microbiota	*Escherichia coli*	Proteobacteria	[67,99]
	*Lactobacillus* spp.	Firmicutes	
Serotonin metabolism	*Pseudomonas putida*	Proteobacteria	[67,99,100]
	*Escherichia coli*	Proteobacteria	
	*Morganella morganii*	Proteobacteria	
	*Klebsiella pneumonia*	Proteobacteria	
	*Staphylococcus aureus*	Firmicutes	
	*Bacillus subtilis*	Firmicutes	
	*Lactobacillus (L. helveticus*	Firmicutes	
	*L. plantarum)*		
	*Enterococcus (E. faecalis, E.* spp.*)*	Firmicutes	
	*Lactococcus lactis* subsp. *cremoris*	Firmicutes	
	*Lactococcus lactis* subsp. *lactis*	Firmicutes	
	*Streptococcus* spp.	Firmicutes	
TMA-producing microbiota	*Desulfovibrio desulfuricans*	Proteobacteria	[87,101,102]
	*Proteus mirabilis*	Proteobacteria	
	*Edwardsiella tarda*	Proteobacteria	
	*Klebsiella pneumonia*	Proteobacteria	
	*Acinetobacter* spp.	Proteobacteria	
	*Citrobacter* spp.	Proteobacteria	
	*Escherichia* spp.	Proteobacteria	
	*Anaerococcus hydrogenalis*	Firmicutes	
	*Clostridium asparagiforme*	Firmicutes	
Tryptamine producing microbiota	*Ruminococcus* spp. *(R. gnavus)*	Firmicutes	[21,77,99,103]
	*Clostridium sporogenes*	Firmicutes	
	*Faecalibacterium prausnitzii*	Firmicutes	
	*Blautia* spp.	Firmicutes	
	*Lactobacillus* spp.	Firmicutes	

This table has been organized following the classical taxonomy classification to facilitate the connection with the previous studies. Here we present the equivalences of the phyla according to the recent actualization performed by the International Committee on Systematics of Prokaryotes (ICSP) [104]: Firmicutes = Bacillota; Bacteroidetes = Bacteroidota; Proteobacterias = Pseudomonadota; Verrucomicrobias = Verrucomicrobiota; Actinobacterias = Actinomycetota.

## 3. Microbiota–Gut–Brain Axis: Linking Peripheral and Central Inflammation

**Figure 1:**
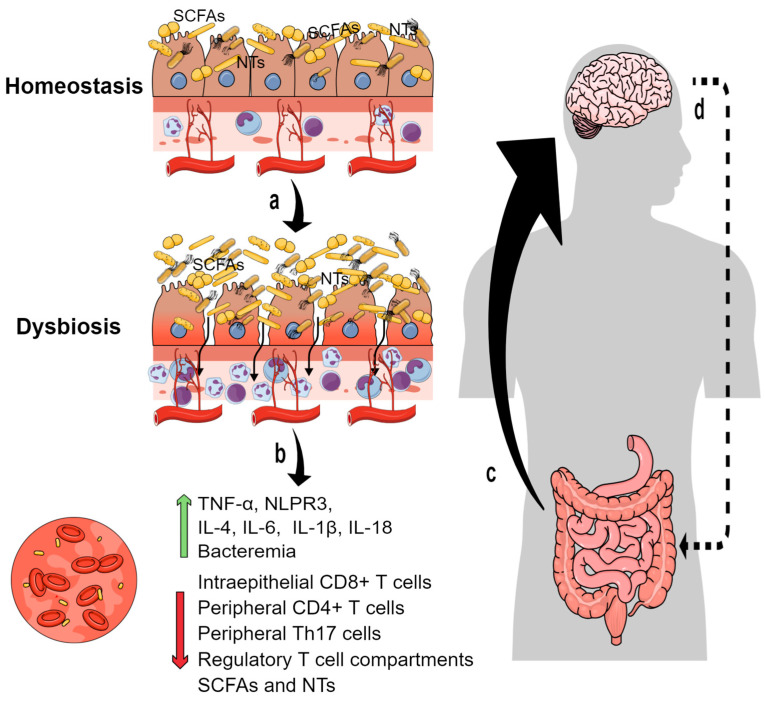
Local and systemic immunological events involving the microbiota–gut–brain axis. (**a**) Under normal conditions, gut displays a homeostatic equilibrium between microbiota and enteric immune cells. Balanced commensal microbiota regulate the levels of neurotransmitters (NTs), such as serotonin or GABA, and neuroprotective substances including some short-chain fatty acids (SCFAs) such as butyrate. (**b**) Dysbiosis or inflammatory processes may lead to the loss of interepithelial junctions between the enterocytes, resulting in a compromised intestinal barrier and a decrease in intraepithelial CD8+ T cells together with an increase in neutrophil and macrophages. Locally, the production of proinflammatory cytokines (TNF-α, IL-6, etc.) is augmented, some of which are involved in NLPR3 inflammasome activation, such as IL-1β or IL-18. Moreover, a decrease in CD4+ and regulatory T and Th17 cells populations takes place. SCFA and NT levels are also altered under dysbiosis, leading to other detrimental consequences within gut and CNS. (**c**) Bacteremia and the release of proinflammatory cytokines, microbial antigens, and toxins into the circulation may be involved in the disruption of the blood–brain barrier, reaching the brain with the consequent impact on glial cells and neuroinflammation. (**d**) In turn, some authors suggest that microbiome dysregulation may happen as a result of neurodegenerative pathological conditions as well.

The MGB axis involves different biochemical and cellular pathways, including the interaction between the microbiome and the immune system [26,27,28]). In fact, the gastrointestinal tract of mammalians is highly enriched in immune cells, which are engaged in a complex dialogue with the gut microbiota. The main components of the intestinal innate immune system are Paneth cells, dendritic cells, neutrophils, macrophages, NK lymphocytes, and mast cells. Interestingly, the immune system enables a symbiotic relationship with the commensal microbiota by maintaining non-inflammatory homeostasis. Most of the innate immune responses are mediated by pattern-recognition receptors such as endosome toll-like receptors (TLRs) or cytosolic nucleotide-binding oligomerization domain (NOD)-like receptors (NLRs), which recognize pathogen-associated molecular patterns (PAMPs) expressed by gut microbiota. Indeed, enteric bacteria can modulate innate immune/inflammatory responses via TLRs and/or NLRs, thus regulating microbe-host interactions and immune tolerance [105]. Actually, several studies have provided examples showing that gut microbiota might modulate innate and adaptative immune responses at the mucosa surface during infection, inflammation, and autoimmunity [26,27]. This state of tolerance depends on additional mechanisms, such as the physical mucus barrier avoiding the contact of microorganisms with epithelium and the secretion of antimicrobial proteins and immunoglobulin A (IgA) [105,106]. In fact, the absence of IgA leads to a strong expansion of anaerobic bacteria, especially mucosa-adherent segmented filamentous bacteria of the phylum Firmicutes [107]. Moreover, epithelial cells are held together by tight junction proteins including claudins, occludins, junctional adhesion molecules, and tricellulin. Notably, pro-inflammatory cytokines such as Tumor Necrosis Factor alfa (TNF-α), IL-4, IL-6, and IL-13 are known to increase the permeability of intestinal epithelium, associated with an up-regulation of claudin [108]. Overall, the dialogue between gut bacteria and the local immune system, together with the proper maintenance of intestinal epithelial barrier, contributes to avoiding the onset of peripheral inflammatory processes (Figure 1). 

Within the CNS, microglia and astrocytes are the essential cells of the innate immune system, performing a myriad of supportive and defensive functions. Just to mention a few, microglia are brain-resident macrophages, support immune surveillance, and participate in synaptic pruning (for review see [109]). For their part, astrocytes maintain synaptic and neuronal homeostatic conditions, modulate synaptic transmission, and are involved in the constitution and maintenance of the BBB [110,111,112], among other functions. The activation of glial cells together with an increased expression of pro-inflammatory mediators are considered central histopathological hallmarks of many neurodegenerative diseases, including AD, PD or Huntington’s disease (HD) [113,114,115,116,117]. Indeed, proinflammatory cytokines, such as IL-1β and TNF-α, are known to favor the cognitive decline and the pathological progression of AD [115,118]. Not only glia but also perivascular myeloid cells have been involved in neuroinflammation [119]. Nevertheless, the role of all these cells in AD is currently under debate, since it is still unclear whether they act as triggers or in the progression of AD pathogenesis, either independently or in combination with Aβ and/or tau. In the early stages of AD, an acute inflammatory response is orchestrated as a protective mechanism, aimed at eliminating the pathological proteinopathies. However, the failure of resolution leads to a chronic toxic activation of the proinflammatory mechanisms, damaging synaptic contacts and neurons. In addition, microglial senescence and/or dysfunction seem to be involved in the onset/progression of this disease [115,120]. Significantly, the microbiome can impact directly or indirectly on glial cells since microbiota are effective for microglia maturation and function [51]. In addition, tryptophan can be metabolized by gut microbiota generating aryl hydrocarbon receptor (AhR) agonists, which modulate astrocyte-mediated neuroinflammation [121]. 

Multiple studies indicate that the central immune system is affected by systemic infections (priming), signals from the peripheric immune system, and the chronic age-associated low-grade inflammation known as *inflammaging* (for review, see [122,123,124]). In fact, the increase of pro-inflammatory factors in the CNS correlates with elevated levels of some cytokines (IL-6, TNF-α, IL-1β, IL-12, IL-18, etc.) in the peripheral blood of AD patients compared with control subjects [125,126] (Figure 1). Fecal microbial transplantation (FMT) from 5xFAD mice into normal C57BL/6 mice gave rise to increased levels of proinflammatory cytokines (TNF-α, IL-1β) in colon, plasma, and brain, reduced hippocampal neurogenesis, and produced memory impairment [127]. Therefore, gut microbiome-immune axis alterations may result in systemic inflammation impacting on glial cells, and consequently, triggering or worsening ongoing neuroinflammatory processes (for review, [128]).

### Microbiota–Gut–Inflammasome Brain Axis 

The inflammasome is one of the most important multimolecular immune pathways involved in CNS homeostasis and neuroinflammation. This signaling can be activated under the presence of certain bacteria and/or their metabolites, constituting a mechanistic link between peripheral and central inflammation. In fact, there is evidence correlating the bacterial gut influence on the inflammasome during CNS inflammation and related pathologies [129]. Many distinct inflammasomes have been identified, each one differentiated by their unique activators from the family NLR/ALR (nod-like receptor/Absent in Melanoma 2-like receptor), and caspase effectors. The canonical inflammasome activation is induced by the proteolytic enzyme caspase-1 [130]. Caspase-1 activates the pro-form of IL-1β, and it has been implicated in the response to amyloid deposition in AD [131]. 

The nod-like receptor pyrin containing 3 (NLRP3) inflammasome is the most representative agent of the NLR receptor family and is widely expressed in immune cells [132]. It has been implicated in several chronic inflammatory diseases and participates in the aggregation of proteins, including Aβ. NLRP3 inflammasome promotes the maturation and secretion of IL-1β in some neurological diseases such as PD or AD [113,132,133]. A higher expression of IL-1β and IL-18 has been reported in microglia, astrocytes, neurons surrounding Aβ plaques, and plasma from AD patients [134,135]. The up-regulated expression of NLRP3, apoptosis, caspase-1, caspase-5, IL-1β, and IL-18 was additionally found in the PBMCs of AD patients [136]. In 2013, Heneka et al. [113] assessed the contribution of the NLRP3 inflammasome to the pathogenesis of AD using the APP/PS1/NLRP3-/- mice and found that caspase-1 cleavage was absent. As expected, aged APP/PS1 mice exhibited severe deficits in spatial memory whereas APP/PS1/NLRP3-/- mice were protected from this cognitive impairment.

In this context, there is evidence supporting the occurrence of dynamic interplays between the gut microbiota and NLRP3 inflammasome (currently called the microbiota–gut–inflammasome–brain axis) [137]. Thus, microbiota modulate peripheral inflammatory signals contributing to brain homeostasis [138]. More efforts are needed to increase knowledge concerning the role of NLRP3 inflammasome activation influenced by gut microbiota in Aβ-mediated inflammatory responses and the development of AD. Therefore, inflammasome or inflammasome-derived cytokines might be considered as therapeutical targets for neurodegenerative conditions.

## 4. Age-Associated MGB Axis Dysfunction

Some of the chronic non-communicable diseases affecting the developed society have been associated with gastrointestinal dysbiosis [139]. As commented, this unbalance may trigger the disruption of the epithelial intestinal barrier and, consequently, some microbial components and products (toxins, amyloids, LPS) could first cross the intestinal mucosa and then the BBB, eventually reaching the CNS [140]. Some of these microbiome changes are initiated because of the normal aging, including a decrease in symbiotic taxa and beneficial bacteria (such as *Akkermansia* or *Bifidobacterium*), along with a progressive enrichment of subdominant families and opportunistic pathobionts, as reported in studies performed over seniors and centenarians [141,142,143]. In this sense, Cryan’s group demonstrated that old (20–21 months) wild-type (WT) mice presented differences in caecal microbiota composition in comparison to that of young (2–3-month-old) mice. Importantly, this age-related shift was associated with increased gut permeability, peripheral inflammation (higher plasma levels of IL-1β and TNF-α), and cognitive damage [144]. In addition, in this same work, they reported a positive correlation between anxiety-like behavior and the increase in Porphyromonadaceae in the aged group. Intriguingly, Li et al. [145] evidenced the relationship between a microbiome shift and the development of mild cognitive impairment (MCI). 

Aging itself contributes to increasing the vulnerability to develop gastrointestinal disorders/dysbiosis by additional and non-mutually exclusive age-related processes. Increased colonic permeability has been associated with elderly people. The remodeling of the epithelial tight junction proteins at this age [146,147] may facilitate the entrance of foreign antigens and microbes to the circulatory system, crossing the BBB and affecting CNS homeostasis. Further studies using mouse models have demonstrated that aging also alters intestinal smooth muscle contractibility as well as neural innervations [100]. Finally, inflammaging, characterized by a chronic low-grade inflammation and the malfunctioning of the adaptative and innate immune system (leucocytes, inflammasome, etc.) because of chronological aging, has been suggested to play a part in MGB axis dysfunction [123]).

## 5. Dysbiosis and Alzheimer’s Disease

A growing body of evidence suggests that alterations in the MGB axis are linked to the development of AD, PD, HD, and amyotrophic lateral sclerosis (ALS), among others [140,148]. Both AD patients and mouse models exhibit dysbiosis with secretion of LPS and toxins leading to leaky gut, which, in turn, may be indirectly involved in the neurodegeneration typical of this pathology [149,150]. Consequently, the impact of dysbiosis and peripheric inflammation over cerebral amyloidosis and neuroinflammation and the involvement of microbiota in the cerebral accumulation of Aβ have been gaining interest in the last decade [151,152,153]. Therefore, a deep understanding of the mechanisms underlying age-related microbiome remodeling would help to develop novel therapeutical strategies to preserve a healthy MGB axis in the context of neurodegenerative diseases.

Many works have detected gut microbiota alterations in MCI [145,154] and AD patients [41,155,156,157] with respect to healthy controls. A recent systematic review and meta-analysis study indicated that there is more abundance of Proteobacteria, *Bifidobacterium* and *Phascolarctobacterium*, but less abundance of Firmicutes, Clostridiaceae, Lachnospiraceae, and Rikenellaceae, in AD subjects in comparison to controls [158]. Generally, these studies showed a decreased fecal bacterial diversity, along with a higher ratio of potential pathogens with respect to beneficial microbes. For instance, a decrease in the abundance of Firmicutes accompanied by an increase in Bacteroidetes has been reported [128]. In this same study, a higher abundance of pro-inflammatory taxa (*Escherichia* and *Shigella*), together with a lower abundance of an anti-inflammatory microbiota (*Eubacterium rectale*), was detected in Aβ-positive patients in comparison to Aβ-negative subjects [128]. A decrease in butyrate-producer bacteria has also been found in AD patients. For instance, plasma circulating markers of endothelial dysfunction positively correlated with the concentration of acetate, valerate, and proinflammatory cytokines, whereas they correlated negatively with the levels of butyrate [157]. Cattaneo et al. (2017) [41] reported a decrease in *E. rectale* in AD patients with cognitive impairment and amyloidosis. Thus, longitudinal studies using senescence and transgenic animal models should be helpful to unravel the basics of age- and AD-associated MGB alterations, as well their future impact on the susceptibility to develop neurodegenerative disorders in the elderly.

## 6. Analyzing Dysbiosis in Transgenic Mouse Models of AD

There is still a long way to go in deciphering the basic mechanisms linking dysbiosis and AD, but considerable evidence points to an intricate connection between microbiota unbalance, peripheral and central inflammation [128]. Indeed, much of our understanding concerning microbiota, immunopathology, and neurological diseases derives from animal models. Specifically, GF models display a variety of intestinal immune defects, including a rare development of gut-associated lymphoid tissues (GALTs), a lower amount of secreted Igs, and reduced intraepithelial CD8+ T cells [27]. GF mice also exhibit a decrease in the total number of peripheral CD4+ T cells, including Th17 cells [29] and Treg compartments [29,159]. 

Indeed, microbiome sequencing of AD models has been progressively incorporated into neuroscience research [160,161]. In this sense, there are several generations of transgenic murine models that may be considered as useful tools for proof-of-concept studies [64,66,162,163,164]. In the mid-1990s, the discovery of inheritable mutations provoking the familiar form of AD (FAD, or early-onset AD) allowed for the development of transgenic mouse models of this disease [165]. The first generation was based on the overexpression of mutated amyloid precursor protein (APP) and/or Presenilins 1 or 2 (PS1, PS2). Most of the monogenic and bigenic APP-based models reproduce, to a greater or lesser extent, the main histopathological events of AD, such as plaque deposition, neuroinflammation mediated by microglia and astroglia, synaptic damage, neuronal loss, and cognitive impairment (for review, see [66,163,166]. Nevertheless, even though it was even possible to replicate the hyperphosphorylation of tau in some amyloidogenic models, it was necessary to introduce *MAPT* mutations associated with primary tauopathies to obtain NFTs. This approach gave rise to Tau-based murine models, which were crossed with amyloidogenic mice leading to novel bigenic (APP/Tau), and trigenic (APP/PS1/Tau) AD models. These animals were able to reproduce both plaques and tangles together with the rest of the typical features of AD [162,167]. At this point, it is important to highlight that to date, there are no reports of *MAPT* mutations in AD patients. Despite the fact that classic transgenic models have functioned as valuable tools to investigate the basic pathogenesis of AD, they are not exempt from artifacts and limitations in reproducing the sporadic form of the disease, the late-onset AD (LOAD). In an effort to overcome these difficulties, new strategies, including the generation of knock-in AD mice, were developed [66,164,168], along with the consortium The Model Organism Development and Evaluation for Late-onset Alzheimer’s Disease (MODEL-AD; https://www.model-ad.org/ (Accessed on 17 April 2023)) [169]. However, this point goes beyond the aim of this work, and it has been discussed in detail in a recent work [164]. Importantly, new modeling approaches combining classical, knock-in, or new generations of AD mice with systemic age-related alterations will help to provide novel insights into the basics of this disease.

An increasing number of studies have primarily focused on characterizing the gut microbiome from classic APP- and tau-based models along aging, or under diverse experimental conditions [161] (Table 2). Many of these works have analyzed the age- and genotype-related changes in the gut microbiome and their impact on cerebral amyloidosis and neuroinflammation. Old APP/PS1 exhibited a lower microbiota biodiversity in comparison to young mice, with an overrepresentation of taxa with proinflammatory activity (such as Verrucomicrobia, *Escherichia/shigella,* Proteobacteria, and *Pseudomonas aeruginosa*) and a decreased abundance of anti-inflammatory bacteria (such as *Eubacterium hallii*, *Bacillus fragilis*, *Bacteroides fragilis*, *Eubacterium rectale*, *Faecalibacterium prausnitzii*, and *Bifidobacterium*) [170]. Concomitantly, these mice exhibit a dramatic increase in gut mucosa permeability. Therefore, these proinflammatory features might influence and promote neuroinflammation, worsening cerebral amyloid deposition [171]. Chronic intraperitoneal injection of *E. coli* LPS increased Aβ burden and gliosis in the APPswe model [172]. Studies using GF or antibiotic-treated APPswe/PS1(L166P) showed a lower Aβ burden in bacteria-depleted mice in comparison to control transgenic animals [173]. Moreover, APP/PS1 mice exhibited significant microbiota changes with aging in comparison to age-matched WT mice. For instance, 8-month-old mice showed a reduction in Firmicutes, Verrucomicrobia, Proteobacteria, and Actinobacteria, together with an increase in Bacteroidetes and Tenericutes phyla. Interestingly, the colonization of the GF-APP/PS1 with APP/PS1 microbiota increased cerebral amyloidosis compared with those receiving microbes from WT counterparts. In young 5xFAD mice (2 months of age), Bacteroides, Firmicutes, and Verrucomicrobia were the three most abundant bacteria at the phylum level. However, at 7 months of age, Firmicutes became the predominant phylum, while the abundance of Bacteroidetes and Verrucomicrobia markedly decreased [174]. In another work, the fecal microbiome of an APPswe/PS1dE9 transgenic mouse model changed at 8–12 months of age in comparison to that of a WT mouse model [175]. These mice exhibited an increase in Verrucomicrobia and Proteobacteria, whereas *Ruminococcus* and *Butyricicoccus* decreased. The functional analysis showed metabolic alterations that may contribute to the increase in amyloid deposition. The abundance of the butyrate-producer *B. pullicaecorum* was reduced, and consequently, the concentration of this SCFA was diminished in feces and brain of these transgenic mice. In addition, this model displays amyloid deposition and ultrastructural abnormalities in the intestine at 5 months of age. In another APP/PS1 mouse model, the reduction in butyrate and acetate levels correlated with a poorer cognitive performance, whereas the levels of propionate were higher than in control mice and correlated with cognitive impairment [176]. Among other mechanisms, butyrate has also been related to epigenetic modulation through the inhibition of histone deacetylase activity. Treating the APP-based mouse model Tg2576 with 4-phenylbutyrate led to a decrease in brain histone acetylation, accompanied by an improvement of cognitive deficits and a reduction in phospho-tau levels [177].

Microbiota modifications in mice can directly or indirectly interfere with the neuroimmune system, altering glial morphology and function. Gliosis and amyloid deposition were modulated by the deleterious effect of antibiotics on the APP/PS1dE9 mouse model gut microbiome [178,179]. APPPS1-21 mice treated with antibiotics showed reduced Aβ burden and altered microglial morphology. Moreover, age-matched antibiotic-treated mice receiving FMT from control APPPS1-21 animals partly restored amyloidosis and microglial phenotype [180]. Frequent FMT from WT donors for 4 months rebalanced colonic gene expression and improved memory deficits, amyloidosis, tau pathology, and gliosis in the receptor ADLP^APT^ mice [181]. These studies support the existence of a connection between the gut microbiota composition and amyloid deposition mediated by glial cells. In addition, studies using 5xFAD mice have evidenced age-associated changes in fecal microbial composition and their repercussion on the immune system and neuroinflammatory markers. At early stages (2–3 months of age), both pro-inflammatory and anti-inflammatory microglia expanded. However, in the following months, only the pro-inflammatory subset continued increasing within the brain, recruiting Th-1 CD4+ cells and promoting proinflammatory microglial activation [174]. As commented, NLRP3 inflammasome seems to participate in the connection between dysbiosis and neuroinflammation. FMT from AD patients to APP/PS1 mice elevated NLRP3 inflammasome and inflammatory factors expression in gut and led to the increase in this marker together with the microglial activation in the hippocampus [182]. Overall, these experiments shed light to the crosstalk between microbiome, peripheral, and central immune systems, suggesting that changes in gut microbiota provoke a shift in the CNS immune profile with the consequent neurological changes [160,174]. 

Nevertheless, there still are conflicting results in the field of microbiome and animal models. For instance, it has been reported that Firmicutes decrease and Bacteroidetes increase in the APPswe/PS1dE9 mouse models in comparison to WT mouse models [173,175], whereas the other way around was found in other studies [171,183]. These differences might be explained by the lack of uniformity in sampling, processing, and sequencing methods or the age, sex, and strains of the animals used. Interestingly, some authors describe that the decrease in amyloid levels or the changes in glial cells after the treatment with antibiotics were found in male but not in female mice [178,180]. Another study reported sex-based differences in the gut microbiota from APP/PS1 mice [176]. Overall, these studies highlight the existence of a microbiome sexual dimorphism (microgenderome) [184] that needs to be considered. 

Comparatively, less studies have been performed to analyze microbiome alterations in models of tauopathy. The mouse P301L exhibits microbiome age-associated alterations in diversity and composition, starting at 3 months of age [185]. In these transgenic mice, the phylum Firmicutes was decreased, whereas Bacteroidetes increased. In this work, authors established correlations between tau pathology and specific gut microbiota, for instance, *Streptococcus* or *Lactococcus* display a negative correlation with AT8 staining at 6 months, whereas *Escherichia-Shigella, Bacteroides*, and *Parabacteroides* were positively correlated with pT231 at 10 months. Recently, D’Argenio et al. [186] compared the gut bacterial and fungal content of 3xTg-AD (presenting plaques and NFTs) and WT mice to identify disease-associated signatures. They conclude that *Coprococcus* genus (Firmicutes phylum), involved in carbohydrate fermentation and SCFAs production, was less abundant in the AD model. Accordingly, similar results have been recently reported in seven different brain-related diseases, including AD, PD, schizophrenia, or MDD, among others [187]. Thus, *Coprococcus* reduction is associated with proinflammation and amyloid deposition in both brain and gut.

**Table 2:** Experimental studies supporting the link between microbiome and AD using transgenic classical models. APP, Amyloid Precursor Protein; GF, Germ-Free; IF, intermittent fasting; PS1, Presenilin; FMT, Fecal Microbial Transplantation; SCFAs, Short Chain Fatty Acid. For review, see [161,188].

AD Model	Microbiota Changes/Interventions	AD-like Pathology Gut Alterations	Reference
APP/PS1	Age-dependent decrease in microbiota biodiversity Reduced Firmicutes/Bacteroidetes ratio Increase in proinflammatory bacteria and decreased abundance of anti-inflammatory bacteria along aging	Neuroinflammation and cerebral amyloid deposition Increased gut mucosa permeability	[170]
APP/PS1	Age-related decrease in microbiota biodiversity assessed using bacteria-derived membrane vesicles in blood Increased Firmicutes, decreased Proteobacteria and Bacteroidetes	Neuroinflammation and cerebral amyloid deposition	[171]
APPswe/PS1dE9	Relevant changes at 8–12 months of age compared to age-matched WT: Lower diversity. Decrease in Firmicutes/Bacteroidetes ratio. Increase in *Verrucomicrobia* and Proteobacteria, and decrease in *Ruminococcus* and *Butyricicoccus* Reduction of the butyrate producer *B. pullicaecorum* Three bacterial taxa differentially represented at 8–12 months	Increased cerebral amyloid deposition Amyloid deposition and ultrastructural abnormalities in the intestine Reduced levels of beneficial SCFAs	[175]
APPswe/PS1dE9	Genotype- and sex-based differences in gut microbiota at 6 months of age	Reduced levels of beneficial SCFAs correlated with cognitive deficits	[176]
5xFAD	Age-associated changes in microbiota: Bacteroides, Firmicutes and Verrucomicrobia were the most abundant phyla at 2 months. Firmicutes become predominant at 7 months	Correlation of gut microbiota with immune cell infiltration and microglial activation	[174]
5xFAD	Age-related changes in fecal microbiome composition Increase in Firmicutes/Bacteroidetes ratio (9–18 weeks of age)	Amyloid expression in the intestine	[183]
Tau P301L	Age-related alterations in microbial diversity and composition, from 3 months of age. Decrease in Firmicutes/Bacteroidetes ratio	Positive correlations of phospho-tau marker and *Escherichia-Shigella*, *Bacteroides* and *Parabacteroides* at 10 months	[185]
3xTg-AD	Decrease in *Coprococcus* genus. Increased abundance of *Escherichia-Gisella* and *Barnesiella*	Proinflammation and amyloid plaques in both brain and gut	[186]
APPswe	Induction of microbiota modifications by chronic intraperitoneal injection of LPS from *E. coli*	Increased Aβ burden and gliosis	[172]
APPswe/PS1 (L166P)	GF or antibiotic-treated mice	Lower Aβ burden, increased levels Aβ-degrading enzymes, and reduced neuroinflammation	[173]
APPswe/PS1dE9	Antibiotic treated mice	Reduced amyloid deposition and increased soluble Aβ (only in males). Altered microglial morphology	[178]
APPPS1-21	Antibiotic treated mice	Reduced Aβ burden (only in males) and altered microglial morphology	[180]
APP	High-fat diet decreased microbial diversity, and increased Firmicutes/Bacteroidetes ratio	Increase in proinflammatory cytokines, and induction of anxiety (more severe in females)	[189]
5xFAD	Intermittent fasting (IF) produced an increase in Firmicutes/Bacteroidetes ratio	Improvement of cognitive decline, amelioration of amyloid burden and reactive gliosis in IF- versus ad libitum group	[190]
ADLP^APT^	FMT from WT donors	Improve of memory deficits, amyloidosis, tau pathology and gliosis. Rebalanced colonic gene expression related to intestinal macrophage activity and the circulating blood inflammatory monocytes	[181]
APPPS1-21	FMT from WT donors led to the restoration of gut microbiome after antibiotic administration	Partly restoration of amyloidosis and microglial phenotype	[180]
GF-APP/PS1	FMT led to significant differences in bacterial diversity depending on the donor (conventionally raised APP/PS1 or WT mice)	Increased cerebral amyloidosis compared with animals receiving FMT from WT mice	[173]
APP/PS1	FMT from AD patients	More severe cognitive impairment and increase in neuroinflammation. Higher NLRP3 expression in the gut	[182]

Fewer studies exist involving models of LOAD, the sporadic form of AD. Nevertheless, Apolipoprotein E (ApoE) isoforms have been reported to differentially affect gut microbiome. The model ApoE-TR showed evidence of an interrelation between the main risk factor of LOAD and gut microbiome profiles [16]. Very recently, Holtzman’s group investigated the role of gut microbiota in tauopathy under the influence of ApoE isoforms [191]. To that purpose, they used the P301S model expressing ApoE3 (TE3) or ApoE4 (TE4) isoforms. Mice that were reared under GF conditions or treated with antibiotics showed a reduction in tau pathology, glial reactivity, and neurodegeneration in comparison to conventionally raised animals in an ApoE isoform-dependent manner. Interestingly, the effect of antibiotic treatment was not always detectable on female mice, as in previous examples. 

Overall, research using animal models strongly support the link between dysbiosis, systemic inflammation, neuroinflammation, proteinopathies, and brain damage. Therefore, animal models are valuable tools to assess the impact of immunological alterations or deficiencies in the development of neuropathological processes and to investigate the correlation between intestinal and cerebral territories.

## 7. Modeling the Link between Metabolic Diseases, the Microbiome, and Late-Onset Alzheimer’s Disease 

Compelling evidence has demonstrated the link between AD and metabolic diseases [192]. Growing data indicate that neuroinflammation may be triggered or accelerated by the appearance of concomitant pathologies such as obesity, type 2 Diabetes (T2D), hypertension, dyslipidemia, atherosclerosis, or cardiovascular dysfunction. Other common events as surgical procedures or infections may be involved as well, including the adaptative response [128,193]. Clearly, the prevalence of diabetes and obesity is constantly increasing in western societies. Both pathologies are associated with cognitive impairment in aged people and thus contribute to increasing the susceptibility of suffering AD [194]. Thus, the question now arises as to whether there exists any connection between dysbiosis and LOAD risk factors [11]. 

Both obesity and T2D are characterized by adipose tissue, liver, muscle, and pancreas inflammation. This phenomenon is accompanied by an accumulation of immune cells releasing proinflammatory cytokines, especially IL-1β, IL-6, and TNFα, involved in the pathogenesis of T2D [195]. AD shares many pathophysiological features with diabetes, such as insulin resistance, oxidative stress, or inflammatory pathways activation. In fact, the term type three diabetes (T3D) is accepted for this neurodegenerative disease [196,197]. On the other hand, obesity itself has been linked to an increased risk of dementia, independently of T2D [192]. The chronic increase in free fatty-acid plasmatic levels correlates with inflammation. Moreover, esterified fatty acids can cross the BBB reaching the brain. Mice receiving a high-fat diet (HFD) exhibited an increase in adipose tissue and higher plasma levels of proinflammatory cytokines (IL-6, TNFα, leptin, resistin…), showing defects in insulin sensitivity and neuroinflammation [198]. A high-fat diabetogenic diet promotes AD pathogenesis, mediated by the effect of saturated fatty acids on TLR4 followed by an inflammatory response [199,200]. In fact, TLR4 has been shown to be involved in glial activation in studies using AD mouse models [201,202]. Moreover, the circulating levels of Aβ, glucose, and insulin correlated with cytokine expression in the brain of AD mouse models [203]. In addition, mitochondrial dysfunction and oxidative stress are also present in obesity, T2D, and AD. This mitochondrial damage is related to the formation of inflammasomes, leading to the activation of caspase-1 and the secretion of IL-1β and IL-18 [192].

More recently, numerous studies have evidenced the importance of dysbiosis in metabolic disorders (for review, [139]). In humans, it has been reported that microbiota profile is different in T2D patients versus non-diabetic controls [204]. As mentioned above, the loss of microbiota homeostasis may lead to a leaky gut with the consequent dissemination of inflammatory cytokines to the brain and other organs [149,205]. Therefore, dysbiosis is now regarded as another proinflammatory contributor of interest for metabolic and neurodegenerative diseases. In turn, lifestyle and dietary habits are recognized as relevant factors involved in the onset and development of both metabolic and neurodegenerative pathologies [189,194], and many studies have linked diet and microbiota in the origin and evolution of metabolic disorders. The study of 200 strains of mice determined that a high-fat and high-sugar diet alters gut microbiota despite the genotypic differences [206]. HFD induced intestinal dysbiosis in mice, including decreased diversity and changes in microbiota composition. An increase in Firmicutes/Bacteroidetes ratio was observed in both male and female mice, independently of the genotype. However, differences regarding caecal microbiota at the family level (increase in *Akkermansia* or decrease in Ruminococcaceae) were more significant in TgAPP females. No relevant differences were found in pro-inflammatory markers between male and female WT mice; importantly, the expression of some cytokines was increased in TgAPP mice compared to WT mice, particularly in females [189]. The chronic administration of a HFD to WT and APP mice demonstrated that fat induces anxiety in a sex-dependent manner, being more obvious in male than in female mice. 

In obese mice, the microbiome exhibits an increased capacity to harvest energy from diet, reported by the fermentation of undigested food components leading to increased SCFAs [149]. Significantly, the transference of obese-prone microbiota to GF mice led to the replication of the obese phenotype: weight gain, adiposity, intestinal permeability, and inflammation [149]. Indeed, diet manipulation is already considered a tool for controlling gut microbiota and thus for preventing and modulating the immune and inflammatory response which finally might modify the AD progression [193,207]. Male Wistar rats fed with a medium or HFD developed memory deficits mediated by an increase in hippocampal IL-1β. This effect was prevented by either blocking action of hIL-1 RA (Central IL-1 receptor antagonist) or by a short-term dietary reversal [208]. FMT from HFD to WT mice triggered behavioral alterations associated with neuroinflammation and altered cerebrovascular homeostasis [149]. On the other hand, Firmicutes are related with cardiovascular and T2D risk, and a hypocaloric diet (low fat or carbohydrates) lowered Firmicutes/Bacteroidetes ratio [209]. Finally, recent studies have shown that intermittent fasting might be a promising approach for neurodegenerative diseases, reducing AD-related pathologies and cognitive impairment in some models of this disease. Intermittent fasting improved the learning ability and memory retention in a 5xFAD-mice compared to 5xFAL (*ad libitum* group), showing a significant reduction in Aβ plaque deposition in typical brain regions and the amelioration of reactive gliosis [116,189]. Finally, the administration of antibiotics, probiotics, and prebiotics has been analyzed in obese mouse models and humans with interesting results, showing their ability to modify the composition of intestinal flora and its effects on insulin sensitivity [210,211]. Altogether, these results allow us to establish associations between the inflammatory state, the diet and microbiome with systemic metabolic diseases and AD.

## 8. Oral Microbiome and Alzheimer’s Disease 

**Figure 2:**
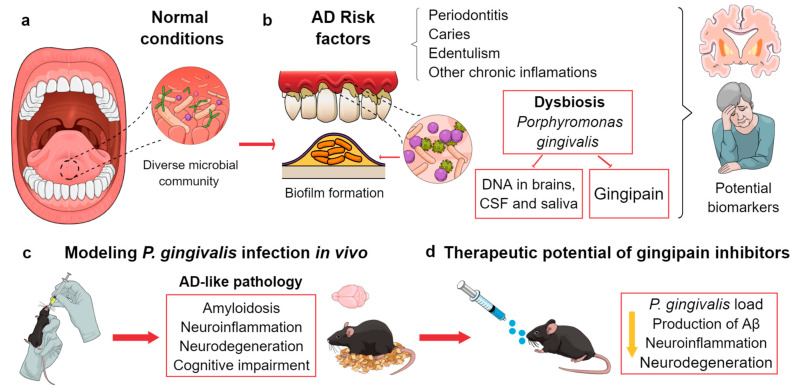
Role of Oral microbiome in Alzheimer’s disease. (**a**) Under normal conditions, the oral cavity displays a healthy homeostatic equilibrium among microbial populations. (**b**) Pathological conditions, such as periodontitis, caries, or other chronic diseases favor the colonization by opportunistic bacteria that produce functional amyloids to create protective biofilms. In this sense, *Porphyromonas gingivalis* infection has been identified as a significant AD risk factor. *P. gingivalis* secretes neurotoxic gingipains, which have been identified in AD samples together with bacterial genetic material. Thus, these molecules might be considered as biomarkers for AD. (**c**) Oral chronic administration of *P. gingivalis* to adult WT mice leads to cerebral AD-like pathology. (**d**) Oral treatment with gingipain inhibitors prevented the neurodegeneration of hippocampal interneurons and reduced amyloidosis and neuroinflammation in previously infected mice. Therefore, these molecules might be a valuable therapeutical approach against *P. gingivalis* brain colonization and the subsequent deleterious effects.

There is growing evidence about the link between oral diseases and AD (Figure 2) [212]. Thus, although this review is mainly focused on the relationship between gut microbiome and AD, it is worth dedicating some paragraphs to the role of oral microbiota in AD patients and its study using animal models. This microbial community is the second most diverse in the human body, with over 700 species identified across individuals. The microbes within the oral cavity include archaea, bacteria, fungi, protozoa, and viruses that play a crucial role in human health and infections [212,213]. The most frequent oral microorganism are bacteria, whose information can be found in The Human Oral Microbiome Database (HOMD) website (http://www.homd.org (Accessed on 10 April 2023)). Even though oral microbiota are personalized, there seems to exist a core microbiome associated to human health [212,214].

As previously described for gut microbiota, the oral microbiome may change in accordance with aging, progressively losing commensal periodontal microbes and thus favoring the colonization by opportunistic bacteria [215]. In fact, caries, edentulism, and periodontitis are common diseases of the mouth cavity in aged people. Moreover, there is mounting evidence suggesting a relationship between oral status, MCI, and AD [212,216,217,218,219]. As mentioned, constant exposure to bacterial pathogenic factors, chronic inflammation, or bacterial brain infection may be relevant AD risk factors. In this sense, periodontitis is a chronic condition leading to the release of pro-inflammatory substances to the systemic circulation, and the emergence of pathogenic species that may cross the BBB, penetrate the CNS, and promote neurodegeneration [220,221,222]. Several bacteria from the oral microbiome, such as the Gram-negative *Porphyromonas gingivalis* (a keystone periodontal pathogenic specie) and *Treponema denticola*, have been found in post-mortem AD samples [223,224,225]. In addition, a higher prevalence of bacteria and antibodies against *P. gingivalis, Actinobacillus actinomycetemcomitans*, and other periodontitis-related bacteria have been detected in brain tissue and serum from AD patients [226,227,228]. Interestingly, the DNA of *P. gingivalis* might potentially be used as a biomarker, since it has been found in the CSF and oral biofluids from living subjects who received a diagnostic of probable AD [224].

Studies with animal models have provided more evidence for the link between periodontal pathogens and AD (Figure 2). Chronic oral administration of *P. gingivalis* provoked AD-like pathology in adult WT mice, reproducing amyloidosis, neuroinflammation, and neurodegeneration [229]. Furthermore, C57BL/6 mice injected with LPS from *P. gingivalis* displayed neuroinflammation and cognitive impairment [230]. In ApoE null mice, periodontal pathogens were reported to access the brain and activate the complement system [225]. More recently, it was shown that periodontitis enhanced systemic and brain inflammation rising the hippocampal levels of IL-1β and TNF-α, worsening AD-like pathology (Aβ and phospho-tau) and cognitive dysfunction in the 3xTg-AD mouse model [231]. 

Importantly, many microbes can produce functional amyloids as a constitutive element of biofilms, a complex protective matrix of bacterial communities. This microbiome-derived amyloid share physical–chemical and immunogenic features with cerebral Aβ and might be able to trigger some proinflammatory AD-related cascades [212,232]. Interestingly, one of the non-pathological functions of Aβ is its antimicrobial activity, linked to its proinflammatory action as a highly conserved effector of the innate immune system [233,234] (for review, see [80,235]) leading to the antimicrobial protection hypothesis of AD. Thus, oral infection may not only lead to the invasion of the brain but also increase the production of Aβ. In this sense, Ishida et al. [236] reported that *P. gingivalis* increased Aβ, TNF-α, and IL-1β levels in an APP mouse model. Surprisingly, chronic systemic injection of *P. gingivalis* in 12-month-old WT mice induced inflammation and Aβ accumulation in liver macrophages/monocytes via cathepsin B/NF-KB signaling, suggesting a contribution to cerebral amyloidosis from the periphery [237]. In addition, toxic proteases produced by *P. gingivalis* (gingipains) have been found to colocalize with Aβ and tau in human AD brains, and their levels correlated with tau and ubiquitin burden [224]. In this same work, it was also described that Aβ42 levels were increased in a periodontitis-induced mouse model. Moreover, gingipains seem to appear before the development of the disease [238]. In this sense, Dominy et al. [224] demonstrated that the administration of small-molecule gingipain inhibitors block GABAergic neurodegeneration in the hippocampal dentate gyrus of orally infected BALB/c mice. Additionally, this treatment not only reduced the cerebral burden of *P. gingivalis* but also decreased the host Aβ response to the infection. Therefore, gingipain inhibitors may be considered a novel therapeutical target to prevent the development or progression of AD in periodontal patients. In fact, there is an ongoing clinical trial in subjects with AD (NCT03823404) with this type of compound.

Overall, in the context of the theories about the infectious origin/comorbidity of AD [239], it is necessary to emphasize the prevention and control of bacterial oral dysbiosis since chronic periodontal diseases and other infections are associated with a higher risk of developing AD [240,241]. In addition, more studies with animal models are needed to evaluate the therapeutic outcome of targeting oral dysbiosis in the framework of sporadic AD.

## 9. Gut Microbiome as a Therapeutic Target for Late-Onset Alzheimer’s Disease

**Figure 3:**
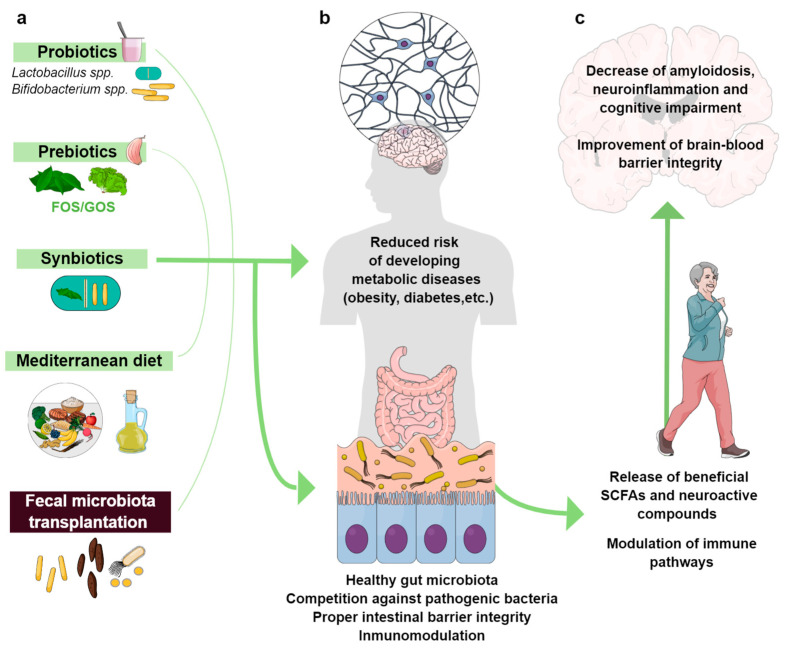
Potential effects of microbiome-based treatments on Alzheimer’s disease patients. (**a**) Several preclinical and clinical trials suggest that the administration of probiotics, prebiotics (fructooligosaccharydes (FOS) and galactans (GOS)), synbiotics, Mediterranean diet, or fecal microbiota transplantation (FMT) may provide benefits to the homeostatic equilibrium of microbiota–gut–brain (MGB) axis, contributing to the prevention or amelioration of neurodegenerative and metabolic diseases. (**b**) A balanced gut microbiota participates in the maintenance of a proper barrier function, reducing intestinal permeability. Moreover, commensal microbes compete for nutrients and space, preventing the invasion of opportunistic pathogens and reducing the release of toxins and proinflammatory cytokines to plasma. (**c**) Healthy microbiota modulates the levels of some neurotransmitters and produces neuroprotective metabolites with positive impact on blood–brain barrier integrity, neuroinflammation and cerebral proteostasis. Overall, these substances may lead to an improvement of cognitive skills under neurodegenerative conditions. Therefore, microbiome-based therapeutic interventions might be considered in the context of personalized medicine.

Given that a healthy nervous system depends on a balanced microbiota, microbiome-based approaches may act as complementary treatments for neurodegenerative diseases, such as AD or PD. Classical interventions include diet and antibiotics [139,242], with direct effects on the gut microbiome. Other possibilities are introducing exogenous bacteria, either directly or indirectly, to manipulate and alter endogenous gut flora in the host. Currently, therapies with probiotic, prebiotic, synbiotic (joint administration of the above) products and FMT, along with changes in the host environment, are assayed as alternatives to classical actions (Figure 3). Indeed, developing preventive strategies based on the enrichment with beneficial bacteria and/or their metabolites may be of great interest once the characteristic profile of the microbiota in AD patients has been established [139,243]. In this sense, the ratio Firmicutes/Bacteroidetes (or Bacillota/Bacteroidota) has been established as a parameter to assess the balance and functionality of the microbiota [139,244]. Under dysbiosis, this ratio is greatly altered, usually due to a significant increase in Bacillota. The mechanisms by which these treatments may elicit beneficial effects include the improvement of the intestinal barrier function, better digestion, absorption and metabolism of nutrients, modulation of gut microbiota composition, pathogen antagonism, and immunomodulation [26,245].

### 9.1. Probiotic Administration as a Treatment for AD

Probiotics are currently defined as “live microorganisms that confer a health benefit when consumed in adequate amounts”, according to the Food and Agriculture Organization/World Health Organization (2002) [246]. *Bifidobacterium* spp., *Lactobacillus* spp., *Lactococcus* spp., *Pediococcus* spp., and other non-pathogenic strains of *E. coli* are commonly used as probiotics, which exert positive effects on the intestinal barrier integrity, reducing inflammatory response and the bacterial translocation [97]. They also compete with pathogenic bacteria for nutrients and binding sites, suppressing bacterial invasion, and blocking pathogen adhesion to epithelial cells [139,245,247].

In recent years, many preclinical studies with probiotics have been developed in animal models of cognitive impairment, aging and AD with promising results [161]. *Lactobacillus pentosus* var. plantarum C29 administration alleviated the D-galactose-induced memory deficits and decreased the expression of inflammatory markers in C57BL/6J mice [248]. Oral administration of ProBiotic-4 (*Bifidobacterium lactis*, *Lactobacillus casei*, *Bifidobacterium bifidum*, *and Lactobacillus acidophilus*) modulated gut dysbiosis and improved cognitive dysfunction in the senescence-accelerated mouse prone 8 (SAMP8) model [249]. Prevention of the cognitive damage induced by intracerebral injection of Aβ was mediated by acetate production after *Bifidobacterium breve* A1 administration. Moreover, gene profiling analysis revealed a reduction of Aβ-induced inflammation in the hippocampus of treated animals [250]. The administration of probiotics accompanied by exercise in APP/PS1 transgenic mice was beneficial for cognitive dysfunction and reduced the hippocampal amyloid burden [251]. A 4-month oral bacteriotherapy with the probiotic SLAB 51 cocktail (*B. longum*, *B. breve*, *B. infantis*, *L. acidophilus*, *L. paracasei*, *L. plantarum*, *L. brevis*, *L. delbrueckii* subsp. Bulgaricus) improved behavioral performance and cortical atrophy in the 3xTg-AD model. Treated animals displayed higher levels of beneficial fecal SCFAs, and a plasmatic profile enriched in anti-inflammatory cytokines along with beneficial hormones such as ghrelin, glucagon-like peptide 1 (GLP-1), and leptin in comparison to control mice [252]. In a later study, 3xTg-AD mice supplemented with the same formulation showed an improvement in cognitive functions, a decrease in amyloidosis and pro-inflammatory cytokine levels, and a sirtuin-1-dependent reduction in oxidative stress [253]. Notably, defects in proteostasis mechanisms (proteasome and autophagy) were partially restored by the treatment, explaining the reduction in amyloid deposition. Therefore, data obtained from different transgenic mice support the use of probiotics as a valid therapeutical approach for managing cognitive impairment, amyloidosis and neuroinflammation in AD. 

In fact, probiotic administration to MCI and AD patients in clinical trials have yielded encouraging outcomes [161,254]. *B. breve* A1 administration to elderly people with memory complaints showed safety and improved their cognitive status [255]. Probiotic mix supplementation (*L. acidophilus*, *L. casei*, *B. bifidum*, and *L. fermentum*) improved insulin metabolism and ameliorated cognitive dysfunction in AD patients [256]. Even though it has been argued that probiotics might induce weight gain in humans, the administration of some strains can produce metabolic benefits and anti-obesity effects. For instance, *L. plantarum* and *L. gasseri* consumption leads to weight loss through the suppression of several hormonal pathways which may function to control some AD-related metabolic alterations [257]. This result has been supported by experiments using transgenic animal models. In fact, a 12-weeks probiotic administration to HFD-fed 3xTg-AD mice not only inhibited weight gain and hypercholesterolaemia, but also reduced cognitive impairment, improved hippocampal spine density, and ameliorated the proinflammatory plasmatic profile in comparison to control HFD 3xTg-AD mice. Authors reported a lower Firmicutes/Bacteroidetes ratio in the treated group [258]. Conversely, the results obtained from different studies with probiotics lack homogeneity, raising doubts about their effectiveness. In fact, the efficacy of probiotic administration can be affected by the dosing, the timing, the proportion of each strain, and the severity of patients, among other factors [247]. Therefore, there is a need for consensus among the protocols for preclinical and clinical assays using probiotics targeting AD, along with considerations regarding the interindividual variability and comorbid conditions.

### 9.2. Prebiotics as a Complement for AD Therapy

Prebiotics are non-digestible food ingredients that are “utilized by host microorganisms conferring a health benefit”. Non-digestible oligosaccharides fructans (fructooligosaccharides (FOS) and inulin) and galactans (galactooligosaccharides or GOS), some types of dietary fiber and sugar alcohols, belong to this conceptual group which has been under constant expansion (for review, see [259]. Prebiotics reach the colon and are fermented by saccharolytic microbes such as beneficial *Bifidobacterium* and *Lactobacillus* [94]. Thus, these compounds impact the composition of the gut microbiota stimulating the growth of beneficiary commensals. 

Prebiotics have demonstrated their capacity to modulate several AD risk factors. For instance, these agents promote intestinal and brain barrier function, modulate the immune response, and improve glucose metabolism [260,261]. *In vitro* supplementation of porcine intestinal epithelial cells with inulin or inulin-derived metabolites produced a butyrate-mediated modulation of intestinal paracellular tight junctions, with a significant upregulation of occludin, claudin-3, and ZO-1 [262]. Prebiotics have also been shown to influence synaptic plasticity, memory function, and brain-derived neurotrophic factor (BDNF) expression in rats, being greater with GOS than with FOS administration. In an Aβ-induced rat model, chitosan oligosaccharides inhibited oxidative stress and neuroinflammatory response, ameliorated neuronal apoptosis, and improved cognitive deficits [263]. GOS and GOS-rich prebiotic treatment have also shown convincing effects on other neurodegenerative diseases. For instance, in a SOD1G93A mouse model of ALS, these prebiotics decreased the activation of astrocytes and microglia as well as motor neuron death [264]. 

Although the translation of these findings to humans looks promising, few studies have examined prebiotics in patients. Moreover, the results are inconsistent; in some studies, the effect is scarce, whereas in others, the prebiotics improved stress and anxiety, as well as mood and recognition memory [260]. Currently, there is only one compound in the AD drug pipeline targeting MGB axis, a prebiotic conceived as a disease-modifier therapy [5]. GV-971 consists of brown algae-derived acidic oligosaccharides aimed to change microbiome to reduce peripheral and central inflammation. The phase III clinical trial will finish by the end of October 2026. Preclinical studies with this compound in APP/PS1 mice indicated that one-month oral administration of GV-971 effectively reconditioned the alterations in gut microbiota, leading to a decrease in infiltrated Th1-cells, neuroinflammation, and AD-like pathology in the brain [174].

### 9.3. Synbiotics and Mediterranean Diet 

It is generally accepted that synbiotics produce better effects over glycemic control and inflammation than probiotics or prebiotics administered alone [139,161,260]. APP-BACE1 *Drosophila melanogaster* receiving the combination of three bioactive probiotics (*L. plantarum*, *L. fermentum* and *B. longum* subsp. infantis) with a polyphenol-rich plant extract showed reduced amyloid pathology and oxidative stress, along with an improvement in acetylcholinesterase activity and insulin-resistance mechanisms [265]. Significantly, some of the reported positive effects were predominant in synbiotic-treated *D. melanogaster* in comparison to the control groups receiving only pre- or probiotics. In this sense, growing evidence supports the beneficial role of polyphenols in preventing and/or delaying AD progression and reducing plaques formation [188]. The increment of these molecules in the colon drives the growth of advantageous microbes such as *Lactobacillus*, and the decrease in pathogenic genera such as *Clostridium*, *Shigella*, and *Escherichia* [266].

On the other hand, healthy eating patterns, characterized by high intake of prebiotics and probiotics in combination with other nutrients, delay cognitive decline and reduce the risk of AD. Substantial evidence highlights that the adherence to the Mediterranean diet (MeDi), enriched in legumes, fruits, vegetables, cereals, polyphenols, non-digestible carbohydrates, and unsaturated fatty acids, is associated with lower rates of cognitive deficits and depression. MeDi reduces the risk of MCI and AD, even frailty in elderly adults [267,268,269,270,271,272]. Many health benefits of MeDi derive from its high content in omega-3 and polyphenols, with anti-inflammatory and antioxidant properties, respectively [273]. In this sense, some groups have analyzed the beneficial impact of olive oil or derivatives in AD transgenic mice with promising results [274,275,276,277]. Furthermore, according to the PREDIMED (PREvencion con Dieta MEDiterranea) study, an intervention with MeDi supplemented with either extra virgin olive oil or mixed nuts is associated with a better global cognitive performance after 6.5 years of follow-up compared with a low-fat control group [278]. DASH diet (Dietary Approaches to Stop Hypertension) shows a very similar composition to MeDi, with more attention on salt introduction (less than 2.4 g/day) [279]. Similarly, MIND diet (Mediterranean-DASH Intervention for Neurodegenerative Delay) consists of a combination of DASH and MeDi diets aimed to slow down neurodegeneration. MIND diet includes the consumption of single dietary components (green leafy vegetables and berries) which have showed a superior effect against cognitive impairment compared to other vegetables or fruits [280].

In addition, MeDi includes a high content in dietetic fiber, which is used by gut microbiota as the main energy substrate. In fact, low-fiber diets results in a decrease in beneficial SCFAs. Therefore, since MeDi is enriched in polyphenols and fiber, the adherence to this diet may help to maintain gut eubiosis [281]. Finally, given the association between AD and gut dysbiosis, MeDi may function as a neuroprotective therapeutic approach not only for modifying the gut microbiota composition and function when needed but also for preventing the development of cognitive impairment [188].

### 9.4. Fecal Microbiota Transplantation

Another therapeutic option for gut dysbiosis is the FMT from healthy donors. There are several procedures available, including fecal enemas, nasoduodenal tubes, colonoscopy administration, or oral capsulated frozen inoculum. FMT is currently the most effective microbiota-based intervention accepted to treat recurrent infections by *Clostridioides difficile* [282]. 

Globally, research testing FMT as a therapeutical means for several neurological diseases have yielded positive results in patients and animal models (for review [283]). In general, as evidenced along this review, studies performing FMT from AD patients or models to GF- or antibiotic-treated animals report a worsening of pathological alterations in the receptors, whereas FMT from healthy donors usually led to amelioration in the receptor group. For instance, in antibiotic-induced pseudo-GF mice, spatial learning and memory improved after receiving feces from senescence-resistant mice (SAMR1), compared to mice whose donor were SAMP8 animals [284]. In this vein, Harach et al., [173] found that FMT from conventionally raised APP mice caused a higher increase in cerebral Aβ pathology than a FMT from WT mice microbiota in a GF-APP transgenic mouse model. Conversely, antibiotic administration to APPPS1-21 mice induced a decrease in Aβ pathology and neuroinflammation, and FMT from control APPPS1-21 to antibiotic-treated APPPS1-21 mice provoked a partial reversion of the initial beneficial antibiotic effect [180]. Specifically involving AD patients, only two case-studies have shown beneficial effects of FMT. Importantly, both patients presented with *C. difficile* infection. In the first report, an 82-year-old AD patient receiving FMT from an 85-year-old woman showed an improvement in cognitive and behavioral symptoms [285]. In the second case, the FMT was carried out from a young healthy man to an aged woman (90 years), who exhibited an amelioration of cognitive skills and microbiota diversity [286]. 

Other studies using animal models highlight the potential of microbiome-based strategies when considering AD as a T3D. FMT from lean donor to male metabolic syndrome subjects improved insulin sensitivity, accompanied by altered microbiota composition in comparison to autologous FMT changes [68]. In another study with diabetic-induced mice, microbiota was different between mice with and without cognitive deficits. Porphyromonadaceae and the genus *Parabacteroides* arose as sensitive indicators of cognitive damage. FMT to pseudo-GF mice demonstrated that the feces from mice without such deficits reversed the cognitive dysfunction in the receptors [287]. At present, the use of FMT has shown encouraging results in animal models of obesity, T2D, and AD regarding cognitive dysfunction and amyloid burden. FMT trials related to human diseases including cancer, neurodegenerative diseases, IBD, and metabolic diseases are still ongoing worldwide [247]. Conversely, FMT carries some concerns about safety, cost, ethical acceptance, and risks including the possible transmission of infectious agents are to be seriously considered [139]. In fact, during the clinical trial AMBITION (NCT03998423) aimed at assessing the impact of FMT in AD patients, the presence of SARS-CoV-2 in fecal material led to the interruption of the study.

## 10. Conclusions

AD still stands as a progressive neurodegenerative pandemic with no cure or a clearly identified etiology. Considering that amyloid- and tau-based therapies are systematically failing, there is an urgent need of developing effective modifying-disease treatments to halt or slow the pace of AD pathology. During the last two decades, the neuroscientific community has been witnessing a paradigm shift since the involvement of the MGB axis has been seriously considered in the context of neurodegenerative and comorbid metabolic diseases. Concomitantly, the crosstalk between peripheral and central immune systems is gaining more and more attention. Thus, AD has moved from being regarded as merely central to a systemic disorder, and consequently, research and therapeutic interventions must be aligned with this complexity. The use of classical and sporadic models of AD under dysbiotic and comorbid conditions is helping to deepen our understanding of the mechanistic basics of this challenging network and to investigate more innovative microbiome-based therapeutic interventions. In this sense, AD treatments will probably include a cocktail of multitarget drugs according to the concept of personalized medicine.

## Data Availability

Not applicable.
